# Shedding of *Trypanosoma cruzi* Surface Molecules That Regulate Host Cell Invasion Involves Phospholipase C and Increases Upon Sterol Depletion

**DOI:** 10.3389/fcimb.2021.769722

**Published:** 2021-10-19

**Authors:** Leonardo Loch, Thiago Souza Onofre, João Paulo Ferreira Rodrigues, Nobuko Yoshida

**Affiliations:** Departamento de Microbiologia, Imunologia e Parasitologia, Escola Paulista de Medicina, Universidade Federal de São Paulo, São Paulo, Brazil

**Keywords:** *Trypanosoma cruzi*, metacyclic trypomastigote, surface molecule shedding, host cell invasion, gp82, gp90

## Abstract

Metacyclic trypomastigote (MT) forms of *Trypanosoma cruzi* have been shown to release into medium gp82 and gp90, the stage-specific surface molecules that regulate host cell invasion, either in vesicles or in soluble form. Here, we found that during interaction of poorly invasive G strain with the host cell, gp82 and gp90 were released in vesicle-like forms, whereas no such release by highly invasive CL strain was observed. Shedding of vesicles of varying sizes by CL and G strains was visualized by scanning electron microscopy, and the protein profile of conditioned medium (CM) of the two strains was similar, but the content of gp82 and gp90 differed, with both molecules being detected in G strain as bands of high intensity in Western blotting, whereas in CL strain, they were barely detectable. Confocal images revealed a distinct distribution of gp82 and gp90 on MT surface of CL and G strains. In cell invasion assays, addition of G strain CM resulted in decreased CL strain internalization. Depletion of gp82 in G strain CM, by treatment with specific mAb-coupled magnetic beads, increased its inhibitory effect on CL strain invasion, in contrast to CM depleted in gp90. The effect of cholesterol-depleting drug methyl-β-cyclodextrin (MβCD) on gp82 and gp90 release by MTs was also examined. G strain MTs, untreated or treated with MβCD, were incubated in serum-containing medium or in nutrient-depleted PBS^++^, and the CM generated under these conditions was analyzed by Western blotting. In PBS^++^, gp82 and gp90 were released at lower levels by untreated MTs, as compared with MβCD-treated parasites. CM from untreated and MβCD-treated G strain, generated in PBS^++^, inhibited CL strain internalization. Treatment of CL strain MTs with MβCD resulted in increased gp82 and gp90 shedding and in decreased host cell invasion. The involvement of phospholipase C (PLC) on gp82 and gp90 shedding was also investigated. The CM from G strain MTs pretreated with specific PLC inhibitor contained lower levels of gp82 and gp90, as compared with untreated parasites. Our results contribute to shed light on the mechanism by which *T. cruzi* releases surface molecules implicated in host cell invasion.

## Introduction

Secreted signaling molecules play essential roles in intercellular communication. As a means of communication between cells, extracellular vesicles (EVs), which are lipid bound particles containing proteins, lipids, and nucleic acids, have gained prominence and are recognized to mediate the regulation of physiological functions and to be involved in pathological processes (Yáñez-Mó et al., 2015; Zaborowski et al., 2015). Among the EV subtypes are exosomes (40–100 nm), formed from multivesicular bodies through the inward budding of the endosome membrane, and microvesicles (100–1,000 nm), released from the cell by the outward budding of the plasma membrane (Mathivanan et al., 2010; Borges et al., 2013; Zaborowski et al., 2015).

Helminths, parasitic protozoa, and bacteria produce EVs (Yáñez-Mó et al., 2015). A recent review on EVs in vector-borne trypanosomatids, *Trypanosoma cruzi*, *Trypanosoma brucei*, and *Leishmania*, has discussed their role in inducing immunomodulatory events and how they affect the parasite interaction with vertebrate and invertebrate hosts (Torrecilhas et al., 2020). As regards *T. cruzi*, the agent of Chagas disease, it was reported three decades ago that the tissue culture-derived trypomastigote (TCT) forms spontaneously shed the entire set of surface polypeptides into the culture medium, mostly as plasma membrane vesicles (Gonçalves et al., 1991). Almost two decades later, it was shown that, upon injection into mice, TCT-shed vesicles increase heart parasitism and generate an intense inflammatory response (Torrecilhas et al., 2009). From then on, an increasing number of studies about *T. cruzi* vesicles have been published, including those related to the insect stage epimastigote and metacyclic trypomastigote (MT) forms. For instance, a proteomic analysis characterized two EV populations and soluble proteins from epimastigote and MT (Bayer-Santos et al., 2013). A study with EVs derived from epimastigotes indicated that they promoted the differentiation of the replicative forms into MTs (Garcia-Silva et al., 2014). In another study, purified EVs from epimastigotes, given to two distinct triatomine insects prior to infection with epimastigotes, affected early parasite migration in the gut of *Rhodnius prolixus* but not in *Triatoma infestans* (Paranaiba et al., 2019). As regards TCT, EVs derived from different *T. cruzi* strains were shown to trigger differential innate and chronic immune responses (Nogueira et al., 2015). Quantitative and qualitative differences in TCT-shed EVs and secreted proteins from different *T. cruzi* strains, revealed by proteomic analysis, were suggested to correlate with infectivity/virulence during the host–parasite interaction (Ribeiro et al., 2018). In Toll-like-receptor 2-transfected Chinese hamster ovary (CHO) cells, an increase in the percentage of TCT-infected cells was observed upon incubation with TCT-shed EVs (Cronemberger-Andrade et al., 2020).

Studies with MTs have revealed that different strains exhibit marked differences in their ability to invade cultured mammalian cells (Yoshida, 2006). A more extensive analysis, using *T. cruzi* strains G and CL, classified as discrete typing unit TcI and TcVI, respectively (Zingales et al., 2009), has shown that the higher efficiency of CL strain in infecting mice, by either intraperitoneal or oral route, and in invading different cell types in culture, is associated with the differential expression of MT-specific surface glycoproteins (Yoshida, 2006) and possibly with their release into medium (Clemente et al., 2016). On the basis that EVs from G strain MTs increased G strain entry into Vero cells but had no effect on invasion by Y strain (TcII), it has been suggested that only parasites of the same classification were capable of modulating the invasion process (Wyllie and Ramirez, 2017). However, this contrasts with the observation that conditioned medium (CM) from G strain, which contains vesicles of varying sizes, significantly inhibited HeLa cell invasion by MTs of either G or CL strain, whereas CM from CL strain had no effect (Clemente et al., 2016). By analyzing the CM of the two strains, as regards the content of MT-specific cell surface glycoproteins gp82 and gp90, which function as a mediator and a downregulator of host cell invasion, respectively (Yoshida, 2006), considerable amounts of these molecules were detected in G strain, as opposed to minimal levels in CL strain (Clemente et al., 2016). The basis for the differential release of gp82 and gp90 by CL and G strain is not known. Here, we analyzed how these molecules are distributed on the surface of CL and G strain MTs and investigated the factors implicated in the shedding process. Experiments were also performed to examine the involvement of gp82 and gp90 contained in G strain CM as inhibitor of host cell invasion by CL strain MTs.

## Materials and Methods

### Parasites, Mammalian Cells, and Invasion Assay

*T. cruzi* strains G and CL were maintained alternately in mice and in liver infusion tryptose (LIT) medium containing 5% fetal bovine serum (FBS). G strain MTs were obtained in high numbers in LIT medium at the stationary growth phase. In the case of CL strain, the parasites were grown for one passage in Grace’s medium (Life Technologies/Thermo Fisher Scientific) to stimulate epimastigote differentiation into MTs. Experiments were performed with MTs purified in a DEAE-cellulose column, as described (Teixeira and Yoshida, 1986). For invasion assay, human epithelial HeLa cells were incubated for 1 h with MTs in Roswell Park Memorial Institute (RPMI) medium supplemented with 10% FBS, at multiplicity of infection (MOI) = 10 (CL strain) or MOI = 20 (G strain), as previously established (Maeda et al., 2012). After fixation in Bouin solution, staining with Giemsa, and sequential dehydration in acetone, acetone:xylol, and xylol, the number of internalized parasites was quantified, by counting a total number of 250 cells.

### Preparation of *Trypanosoma cruzi* Conditioned Medium and Detergent-Soluble Extract

CM was prepared by incubating MTs (5 × 10^8^/ml) for 30 min in RPMI medium containing 1% FBS. After centrifugation at 1,500 *g* for 5 min, the supernatant was collected and filtered in a 0.20-μm syringe filter. The pellet was reconstituted with PBS to the original volume and lysed with 0.5% nonionic detergent Igepal CA630 (USB Corporation). The supernatant, resulting from centrifugation at 14,500 *g* for 10 min, constituted the detergent-soluble MT extract. In some experiments, CM was prepared by incubating MTs in serum-free medium or in PBS^++^ (phosphate-buffered saline containing per liter 140 mg of CaCl_2_, 400 mg of KCl, 100 mg of MgCl_2_·6H_2_O, 100 mg of MgSO_4_·7H_2_O, and 350 mg of NaHCO_3_).

### Visualization of *Trypanosoma cruzi* Metacyclic Trypomastigote and Interaction With Host Cell by Confocal Microscopy

Purified parasites were fixed with 4% paraformaldehyde for 20 min, washed in PBS, and placed onto glass slides and dried. Afterwards, the parasites were incubated for 1 h with antibody directed to MT surface molecule and, following washes in PBS and incubation with the secondary antibody (Alexa Fluor-conjugated IgG), diluted 1:500 in PGN (0.15% gelatin in PBS containing 0.1% sodium azide), plus 10 μM of 4′,6′-1-diamino-2-phenylindole dihydrochloride (DAPI). To examine the host cell–MT interaction, HeLa cells were incubated with parasites for 30 min and then processed for immunofluorescence and confocal microscopy visualization, essentially as previously described (Rodrigues et al., 2017), using rabbit anti-human LAMP2 antibody, mAb directed to gp82 or gp90, Alexa Fluor-conjugated IgG, and DAPI. The coverslips were mounted in ProLong Gold (Invitrogen). Images were acquired in a confocal microscope (Instituto de Farmacologia e Biologia Molecular (INFAR), Universidade Federal de São Paulo), using ×63 objective, processed, and analyzed using Leica LAS AF and Imaris (Bitplane) software.

### Scanning Electron Microscopy

Parasites were washed with PBS, attached to coverslips pretreated with 0.05% poly-l-lysine for 30 min, and washed in water. After 5-min centrifugation at 500 g, the parasites were fixed for 1 h, at room temperature, with 2.5% glutaraldehyde in 0.1 M of sodium cacodylate buffer, pH 7.2. Following four washes with 0.1 M of sodium cacodylate buffer, post-fixation with 1% osmium tetroxide in the same buffer, at room temperature, and washings with cacodylate buffer, the parasites were treated for 30 min with 1% tannic acid in water. After three washes in water, 30-min impregnation with 1% osmium tetroxide, and three washes in water, the samples were subjected to a gradual dehydration in a series of ethanol solutions and dried at the critical point apparatus using CO_2_. After assembly in support of the SEM sample holder (stub) using Superglue, the material was coated with gold by sputtering and observed in scanning electron microscope.

### Immunoprecipitation of Gp82 or Gp90 With Magnetic Beads Crosslinked to Specific Monoclonal Antibody

Protein G magnetic beads (Pierce Crosslink Magnetic IP/Co-IP Kit, Thermo Fisher Scientific), crosslinked with anti-gp82 mAb 3F6, anti-gp90 mAb 1G7, or with mAb 2C2 directed to an amastigote surface antigen, were incubated with G strain CM for 2 h at room temperature, along with the control empty beads. After collection, the CM was checked for depletion of gp82 and gp90 by Western blotting and used for MT invasion experiments.

### Antibodies and Reagents

Alexa Fluor 555-conjugated anti-mouse IgG, Alexa Fluor 488-conjugated anti-mouse IgG, and Alexa Fluor 488-conjugated anti-rabbit IgG were from Thermo Fisher Scientific. Phospholipase C (PLC) inhibitor U73122 and methyl-β-cyclodextrin (MβCD) were from Sigma/Merck.

### Statistical Analysis

Student’s *t*-test (GraphPad Prism software Version 6.01) was employed to evaluate significance between two groups. For multiple comparisons, we used one-way NOVA followed by Bonferroni’s post-hoc test.

### Ethics Statement

All procedures conformed to Brazilian National Committee on Ethics Research (CONEP) guidelines, and the study was approved by the Committee on Ethics of Animal Experimentation of Universidade Federal de São Paulo (protocol number: CEUA 9780200918).

## Results

### During Interaction With the Host Cell, Gp82 and Gp90 Are Released at High Levels by G Strain, But Not by CL Strain

**Figure 1:**
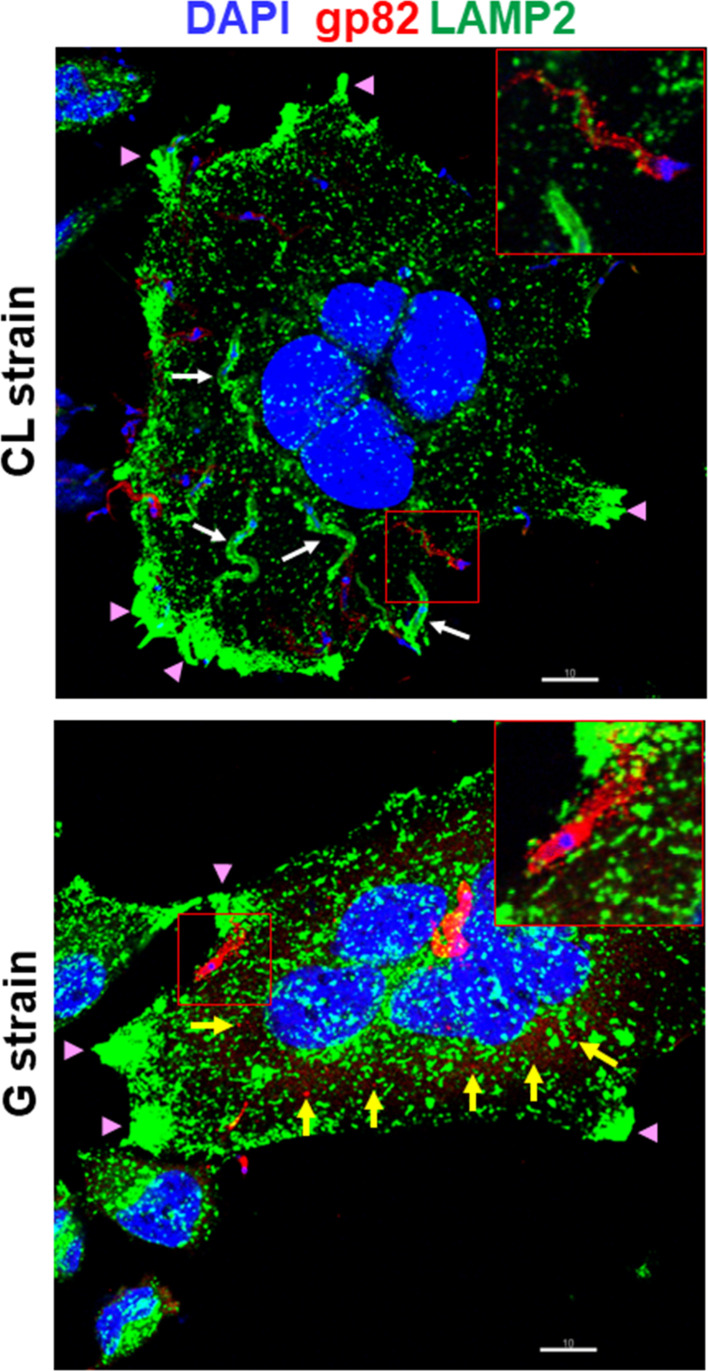
Differential release of surface molecule gp82 by metacyclic trypomastigotes (MTs) of *Trypanosoma cruzi* strains CL and G during interaction with the host cell. HeLa cells were incubated for 30 min with MTs of CL or G strain and processed for immunofluorescence and confocal microscopy visualization of lysosomes (green), nucleus (blue), and gp82 (red). Scale bar = 10 µm. Note the internalized CL strain MTs with lysosome membrane marker (white arrow), the gp82 released by G strain MTs (yellow arrow), and the lysosome accumulation at the cell edges (pink arrowhead). Shown on the upper right side of each panel is the magnified image from the smaller framed area.

**Figure 2:**
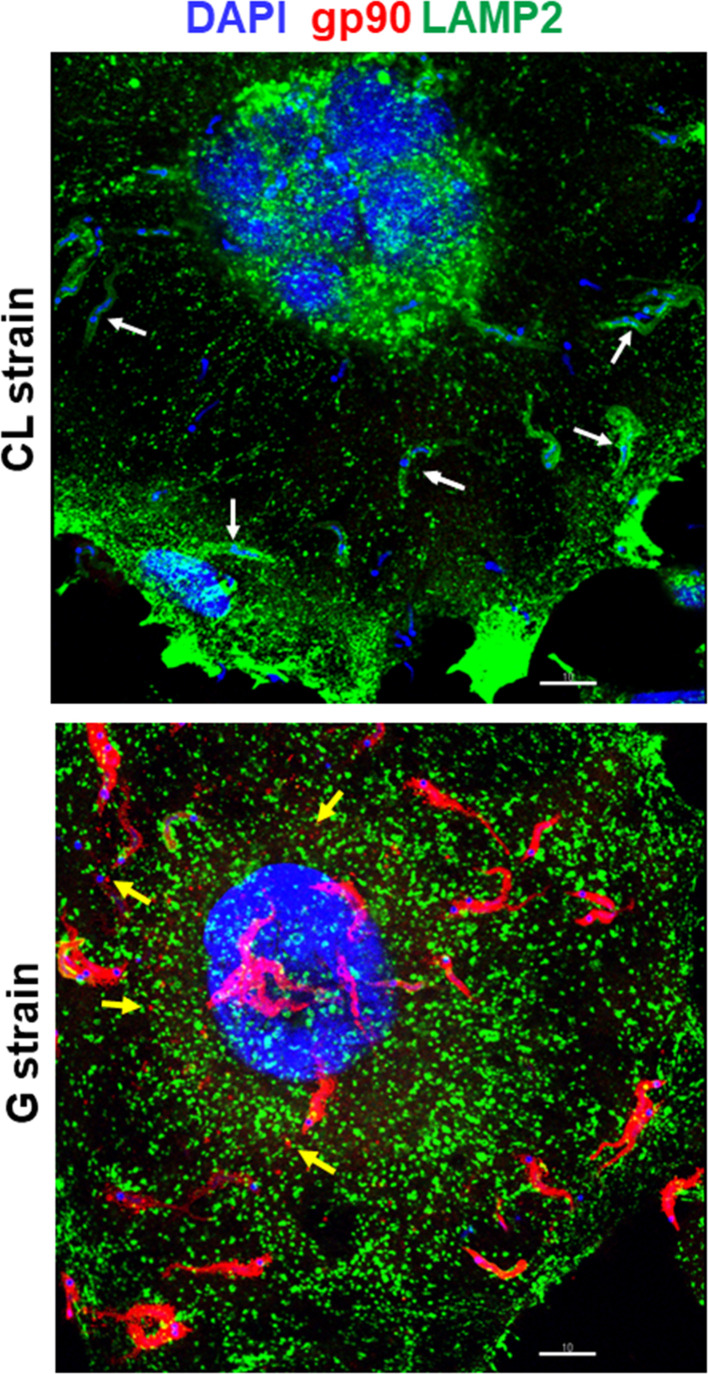
Differential release of surface molecule gp90 by metacyclic trypomastigotes (MTs) of *Trypanosoma cruzi* strains CL and G during interaction with the host cell. HeLa cells were incubated for 30 min with MTs of CL or G strain and processed for immunofluorescence and confocal microscopy visualization of lysosomes (green), nucleus (blue), and gp90 (red). Scale bar = 10 µm. Note the internalized CL strain MTs with lysosome membrane marker (white arrow) and the gp90 released by G strain MTs (yellow arrow).

A previous study has shown that gp82 and gp90 are released into medium in considerable amounts by G strain, either in vesicles or in soluble form, whereas release by CL strain MTs is minimal (Clemente et al., 2016). Here, we performed experiments to examine the shedding of these molecules during MT interaction with the host cell. HeLa cells were incubated for 30 min with G or CL strain MTs and then processed for indirect immunofluorescence, using anti-gp82 mAb 3F6 or anti-gp90 mAb 5E7, and anti-LAMP antibody for lysosome visualization. Upon G strain interaction with cells, gp82 was detected in vesicle-like forms, around the parasite and also attached to cells, or apparently internalized, whereas no such release by CL strain was observed (**Figure 1**). Both G and CL strain MTs induced the spreading of lysosomes and accumulation at the cell edges, an event known to be stimulated by gp82 (Cortez et al., 2016), and was particularly evident in large multinucleated cells (**Figure 1**). These large cells, which represent less than 10% of the total cell population, were more susceptible to gp82-mediated CL strain invasion (**Figure S1**), possibly because they express higher levels of gp82-receptor LAMP2 on the surface (Rodrigues et al., 2019; Onofre et al., 2021). CL strain internalization was evidenced by incorporation of lysosome membrane marker into the parasitophorous vacuole (**Figure 1**). As regards gp90, its release by G strain upon interaction with HeLa cells was similar to that of gp82, whereas shedding by CL strain was not detected (**Figure 2**). In contrast to CL strain, association of G strain with lysosome membrane marker was not seen, even when a high number of adherent parasites were visualized (**Figure 2**).

### Vesicle Shedding and Protein Profile of Conditioned Medium Are Similar in CL and G Strains

**Figure 3:**
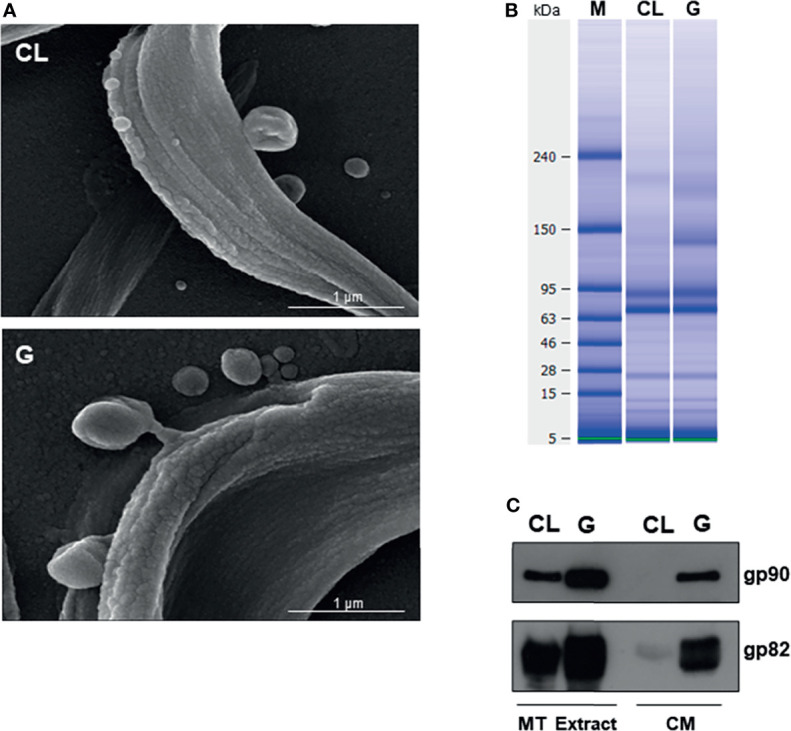
Comparable shedding of vesicles by metacyclic trypomastigotes (MTs) of *Trypanosoma cruzi* strains CL and G and differential release of gp82 and gp90 into medium. **(A)** Parasites were washed in phosphate-buffered saline (PBS) and processed for analysis by scanning electron microscopy. Scale bar = 1 μm. **(B)** Protein profile of conditioned medium (CM) from CL and G strains, revealed by Agilent 2100 Bioanalyzer system. **(C)** Profile of gp82 and gp90 detected in Western blotting of CL and G strain CM and the respective detergent extracts.

Both CL and G strains were found to release comparable numbers of vesicles, with the difference that G strain released EVs of large size in higher amounts and EVs of smaller size in lower numbers, as compared with CL strain (Clemente et al., 2016). We visualized vesicles of varying sizes shed by CL and G strains by scanning electron microscopy (**Figure 3A**). As regards the protein content of CM, previously shown to contain large and small vesicles, as well as soluble factors (Clemente et al., 2016), the analysis performed by using High Sensitivity Protein 250 kit and the Agilent 2100 Bioanalyzer system revealed a similar profile in CL and G strains (**Figure 3B**). In the range of 56.5–124.8 kDa, the 70.5-kDa protein was the most abundant (**Figure S3**). The major difference was observed in Western blotting of CM. Gp82 and gp90 were detected in G strain as bands of high intensity, whereas in CL strain, they were barely detectable (**Figure 3C**), compatible with the result from the immunofluorescence analysis of MT interaction with HeLa cells (**Figures 1**, **2**).

### CL and G Strains Differ Morphologically and in the Expression/Distribution of Gp82 and Gp90

**Figure 4:**
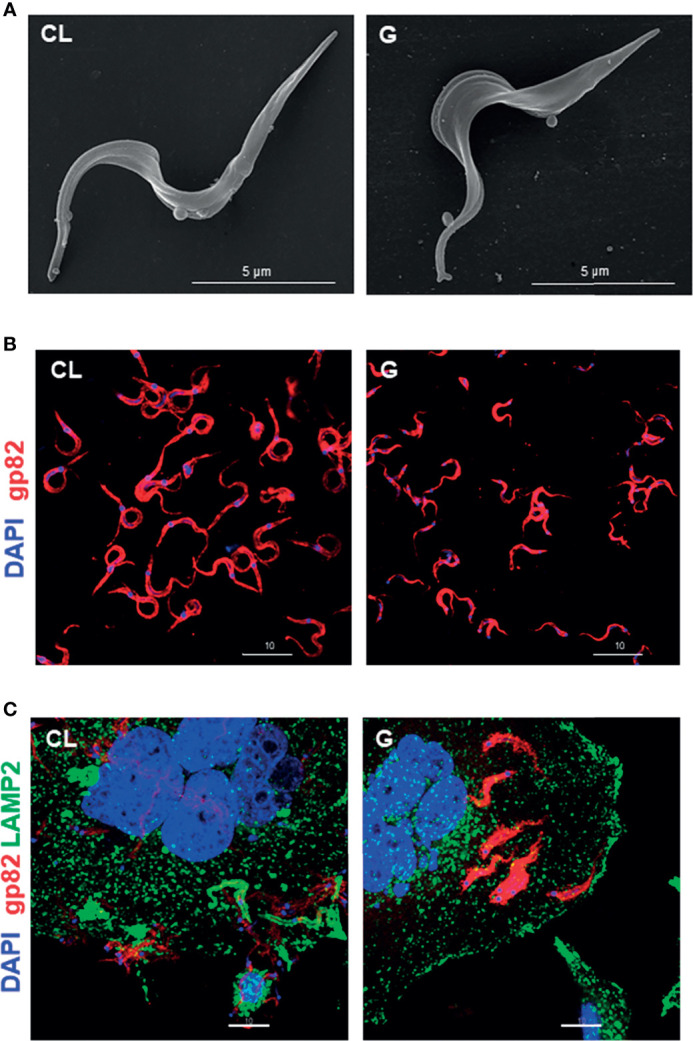
Distinct morphology and pattern of gp82 expression in CL and G strain metacyclic trypomastigotes (MTs) upon interaction or not with HeLa cells. **(A)** Purified MTs were processed for analysis by scanning electron microscopy. Scale bar = 5 μm. **(B)** MTs were processed for immunofluorescence and confocal microscopy visualization of gp82 (red) and nucleus (blue). Scale bar = 10 µm. **(C)** MTs were incubated with HeLa cells for 30 min and processed for immunofluorescence and confocal microscopy visualization of lysosomes (green), nucleus (blue), and gp82 (red). Scale bar = 10 µm. Note a patchy distribution of gp82 on CL strain surface.

**Figure 5:**
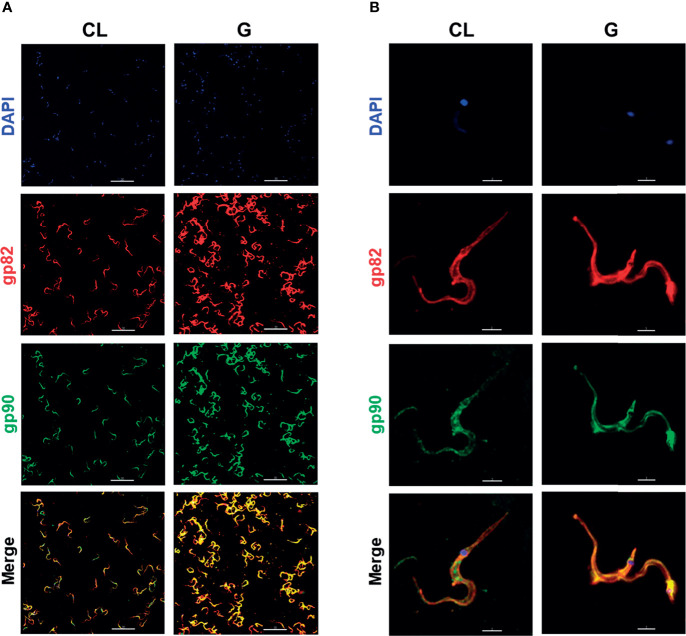
The relative distribution of gp82 and gp90 on metacyclic trypomastigote (MT) surface of CL and G strains. **(A)** MTs were processed for immunofluorescence and confocal microscopy visualization of gp82 (red), gp90 (green), and nucleus (blue). Scale bar = 30 µm. **(B)** Individual parasite is depicted to show the segregated distribution of gp82 and gp90 in CL strain and closer localization in G strain. Scale bar = 3 µm.

Morphology is one of the distinctive features between CL and G strains. MTs of CL strain are slender and longer, as shown by scanning electron microscopy (**Figures 4A** and **S3A**). Such a difference in morphology between strains was evident at the population level, as shown by immunofluorescence, using anti-gp82 mAb 3F6 (**Figure 4B**). By calculating the area of parasites shown in **Figure 4B**, using ImageJ v. 1.53f51, CL strain MTs were found to be significantly larger than G strain MTs (**Figure S3B**). We also noted, particularly in assays of MT interaction with HeLa cells, a distinct pattern of gp82 expression in the two strains, with CL strain exhibiting a patchy gp82 distribution (**Figure 1**, framed area on the upper right side, and **Figure 4C**). To examine the relative distribution of gp82 and gp90 on MT surface, the parasites were processed for immunofluorescence analysis, using anti-gp82 mAb 3F6 and polyclonal anti-gp90 antibody generated in rabbit. Confocal images revealed a segregated distribution of gp82 and gp90 in CL strain, whereas in G strain, the two molecules appeared to be closely localized (**Figure 5A**). This was more evident in the magnified image of an individual parasite (**Figure 5B**). It is possible that the differential distribution of gp82 and gp90 on the surface of CL and G strains influences the differential shedding. Segregated localization of gp82 and gp90 in CL strain could render these molecules less amenable to shedding, whereas their release by G strain would be facilitated by the localization in a microdomain more susceptible to factors involved in shedding mechanism. Gp82 and gp90 appeared to be expressed at higher levels in G strain than in CL strain, as judged by the higher fluorescence intensity (**Figure 5A**), compatible with the Western blotting profile of the MT extract (**Figure 3C**).

### CL Strain Invasion Is Modulated by G Strain Conditioned Medium in a Manner Correlated With Gp82 and Gp90 Levels

**Figure 6:**
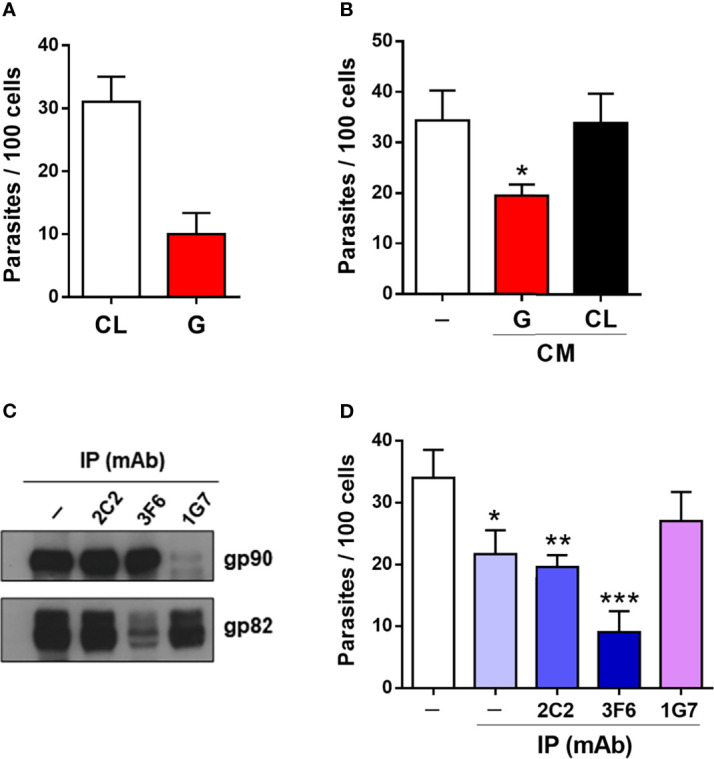
Effect of gp82 and gp90 contained in G strain CM on host cell entry of CL strain metacyclic trypomastigotes (MTs). **(A)** HeLa cells were incubated for 1 h with CL or G strain MTs and processed for Giemsa staining and intracellular parasite quantification. Values are the means ± SD of five independent assays performed in duplicate. **(B)** HeLa cells were incubated for 1 h with CL strain MTs in the absence or presence of CM from G or CL strain and processed for internalized parasite quantification. Values are the means ± SD of five independent assays performed in duplicate. MT invasion was significantly inhibited by G strain CM (*p < 0.001), but not by CL strain CM. **(C)** Western blotting profile of gp82 and gp90 in G strain CM upon 2-h incubation with magnetic beads crosslinked to the indicated mAbs. Note the depletion of gp82 and gp90. **(D)** HeLa cells were incubated for 1 h with CL strain MTs in the absence or presence of CM shown in **(C)**, and the internalized parasites were quantified. Values are the means ± SD of three independent assays performed in duplicate. Significant inhibition by different CMs was detected, except for the CM immunoprecipitated by mAb 1G7 (*p < 0.05, **p < 0.01, ***p < 0.005).

In invasion assays, when HeLa cells were incubated with MTs for 1 h, about threefold higher number of CL strain is internalized, as compared with G strain (**Figure 6A**). The lower invasive capacity of G strain is correlated with the content of gp82 and gp90 at higher levels in CM (**Figure 3C**). It was previously shown that G strain CM can inhibit CL strain invasion (Clemente et al., 2016), but to what extent that effect was due to gp82 and/or gp90 remains to be determined. Experiments were performed to address that question. First, to confirm the inhibitory activity of G strain CM, HeLa cells were incubated for 1 h with CL strain MTs in the presence of G or CL strain CM, at 1:2 dilution, and the number of internalized parasites was quantified. CL strain internalization was significantly reduced by G strain CM, but not by CL strain CM (**Figure 6B**). Next, magnetic beads crosslinked to anti-gp82 mAb 3F6 or to anti-gp90 mAb 1G7 were prepared and incubated for 2 h at room temperature with G strain CM. As controls, beads crosslinked to mAb 2C2, directed to amastigote-specific surface antigen (Andrews et al., 1987), and empty beads were used. Gp82 and gp90 were considerably depleted upon incubation of CM with beads coupled to mAb 3F6 and mAb 1G7, respectively, but not with empty beads or beads coupled to mAb 2C2 (**Figure 6C**). Invasion assays were then performed by incubating CL strain MTs with HeLa cells in the presence of CM prepared as above. Parasite invasion was significantly reduced in the presence of control CMs, an effect that was reversed by gp90 depletion, whereas gp82-depleted CM exhibited a higher inhibitory activity than the control CMs (**Figure 6D**). This finding indicates that gp90 is mainly involved in decreasing MT infectivity, further reinforcing its downregulatory role on host cell invasion. CM derived from G strain upon interaction with target cells was also tested. Parasites were placed onto plates, either uncoated or with adherent HeLa cells. After 30-min incubation, the medium containing parasites was collected and centrifuged. The supernatant was filtered and used for Western blotting and cell invasion assays. Western blotting analysis of CM from MTs that contacted HeLa cells showed a modest increase in the intensity of gp82 of gp90 bands (**Figure S4A**), on the order of 10%, as quantified using GelAnalyzer 19.1 software. Cell invasion assays showed that interaction of G strain with HeLa cells did not generate CM with higher inhibitory activity towards CL strain internalization (**Figure S4B**).

### Shedding of Gp82 and Gp90 Increases Upon Metacyclic Trypomastigote Treatment With Cholesterol-Depleting Drug and Decreases by Treatment With Phospholipase C Inhibitor

**Figure 7:**
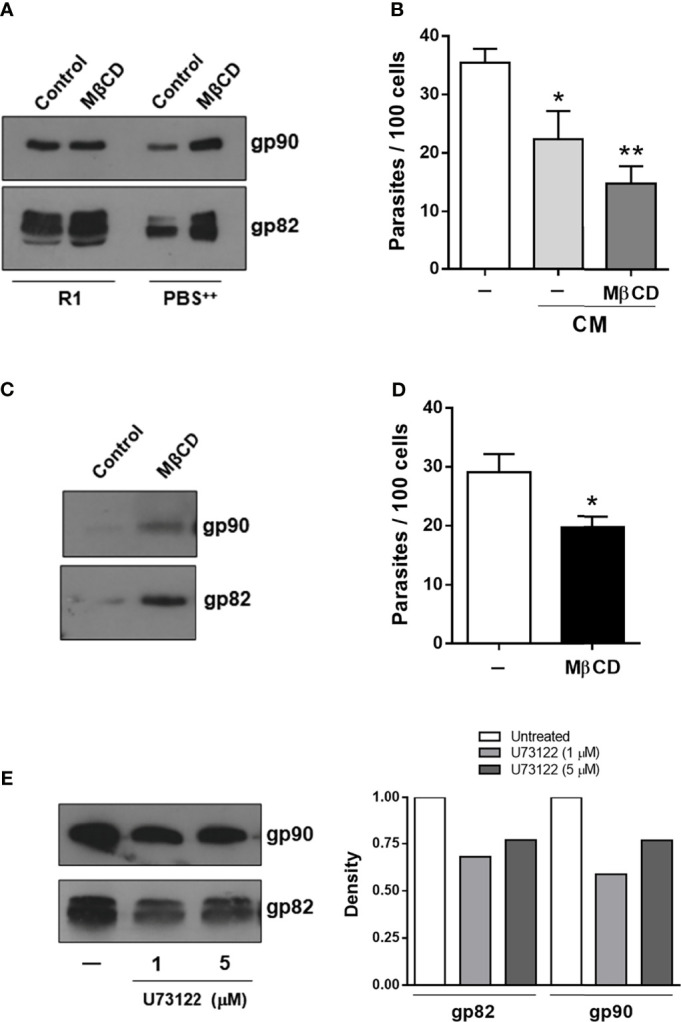
Increase in gp82 and gp90 shedding upon metacyclic trypomastigote (MT) treatment with cholesterol-depleting drug and decrease by treatment with phospholipase C (PLC) inhibitor. **(A)** Untreated and methyl-β-cyclodextrin (MβCD)-treated G strain MTs were incubated in R1 or in PBS^++^ for production of CM, which was analyzed by Western blotting. Note the increased levels of gp82 and gp90 in CM from MβCD-treated parasites, generated in PBS^++^. **(B)** HeLa cells were incubated with CL strain MTs in PBS^++^, in the absence or presence of CM generated by G strain in PBS^++^. Values are the means ± SD of three independent assays performed in duplicate. CMs from untreated and MβCD-treated G strain significantly reduced CL strain internalization (*p < 0.05, **p < 0.001). **(C)** Untreated and MβCD-treated CL strain MTs were incubated in R0 to generate CM, which was analyzed for gp82 and gp90 contents. The levels of both molecules were increased in MβCD-treated parasites. **(D)** HeLa cells were incubated for 1 h with untreated or MβCD-treated CL strain MTs and then processed for intracellular parasite quantification. Values are the means ± SD of three independent assays performed in duplicate. Cell invasion capacity was significantly reduced in MβCD-treated MTs (*p < 0.005). **(E)** G strain MTs were treated with the PLC inhibitor U73122 at the indicated concentrations and then incubated in R1 to generate CM, which was analyzed by Western blotting. Note the decreased gp82 and gp90 levels in CM from U73122-treated parasites, confirmed by measuring the intensity of the bands using GelAnalyzer 19.2 software, as shown on the right.

We examined the effect of cholesterol-depleting drug MβCD on gp82 and gp90 release by MTs. Trypanosomatids synthesize ergosterol-related sterols (Roberts et al., 2003), and treatment of MTs with MβCD has been shown to efficiently deplete ergosterol (Fernandes et al., 2007). Both gp82 and gp90 are anchored to MT plasma membrane by glycosylphosphatidylinositol (GPI) moiety (Yoshida, 2006). GPI-anchored membrane proteins are among those that are targeted to lipid rafts, which are microdomains enriched in cholesterol and sphingolipids. G strain MTs were treated with 10 mM of MβCD for 45 min in serum-free RPMI medium. After removal of the drug, the parasites were incubated for 30 min in RPMI medium containing 1% FBS (R1) or in PBS^++^, which is basically PBS containing Ca^2+^ and Mg^2+^, along with the untreated controls, and the untreated CM generated under these conditions was analyzed by Western blotting. In PBS^++^, gp82 and gp90 were released at low levels by untreated MTs, and at higher levels in MβCD-treated parasites (**Figure 7A**). The effect of these CMs on CL strain invasion was tested. CMs generated in PBS^++^ by untreated and MβCD-treated parasites exhibited significant inhibitory activity (**Figure 7B**). We also examined the effect of MβCD on CL strain. An increase in gp82 and gp90 release was detected in CM generated in R0 by MβCD-treated parasites (**Figure 7C**). HeLa cells were then incubated for 1 h with untreated or MβCD-treated CL strain MTs in R0, and the internalized parasites were quantified. As shown in **Figure 7D**, MβCD-treated parasites exhibited significantly reduced capacity to invade cells. As GPI-solubilizing PLC was found in all G strain developmental forms (de Almeida and Heise, 1993), we tested the involvement of PLC on gp82 and gp90 shedding, and G strain MTs were treated for 30 min with phosphoinositide-specific PLC inhibitor U73122 at 1 or 5 µM. After removal of the drug, the parasites were incubated for 30 min to generate CM. Treatment of MTs with PLC inhibitor decreased the gp82 and gp90 shedding, as confirmed by measuring the intensity of the bands using GelAnalyzer 19.2 software (**Figure 7E**).

## Discussion

Lysosome spreading and exocytosis are critical for the process of *T. cruzi* invasion of host cells (Tardieux et al., 1992; Fernandes et al., 2011; Martins et al., 2011; Cortez et al., 2016). Studies with *T. cruzi* CL strain have shown that efficient entry of MTs into target cells is mediated by gp82, which binds to its receptor LAMP2 and triggers lysosome mobilization to the cell periphery (Cortez et al., 2016; Rodrigues et al., 2019; Onofre et al., 2021). Here, we have found that lysosome spreading is also induced by poorly invasive G strain MTs upon interaction with the host cell, possibly as a result of recognition of shed gp82 by its receptor. Shedding of gp90, the down modulator of MT internalization (Málaga and Yoshida, 2001), was also detected. By contrast, release of gp82 and gp90 by CL strain MTs was barely detectable. Host cell invasion experiments showed the inhibitory effect of G strain CM on CL strain internalization. We examined to what extent this effect was due to gp82 and/or gp90, by depleting CM from these molecules. The inhibitory activity of CM was in the most part reversed by depletion of gp90. On the other hand, depletion of gp82 resulted in an increased inhibitory capacity of CM. From this result, we envisage the possibility that gp82 hampers gp90 interaction with the host cell, so that its absence potentiates the gp90 binding, increasing the downregulatory effect on MT invasion. The inhibitory effect of gp82-depleted CM on CL strain internalization is similar to that observed when HeLa cells were incubated with MTs in the presence of native or recombinant gp90 (Rodrigues et al., 2017).

In our search for factors that influence the release of surface molecules by MTs, the involvement of sterol was investigated. With the use of the antibiotic filipin as a probe, cholesterol was found to be homogenously distributed throughout *T. cruzi* epimastigote plasma membrane (Souto-Padrón and de Souza, 1983) and presumably is distributed in MTs in the same manner. It has been reported that cholesterol depletion by MβCD enhances shedding of cytokine receptor CD30 in lymphoid-derived cell lines (von Tresckow et al., 2004). By examining the effect of MβCD on gp82 and gp90 release by G strain MTs in PBS^++^, we detected an increased shedding of both molecules. As compared with CM generated in PBS^++^ by untreated parasites, the CM from MβCD-treated MTs contained higher gp82 and gp90 levels and, accordingly, exerted higher inhibitory activity on CL strain invasion. Treatment of CL strain MTs with MβCD resulted in increased shedding of gp82 and gp90, although not to levels comparable with G strain, and led to a decrease in the ability to invade HeLa cells. These results suggested that gp82 and gp90 are partitioned, at least partially, into lipid rafts. In G strain, gp82 and gp90 were closely localized on MT surface and were released in medium containing serum, as well as in serum-free medium. Gp82 and gp90 were more segregated in CL strain. Trans-sialidase and mucins, which are also GPI-anchored glycoproteins, were reported to be separately distributed on TCT surface and contained in different and highly stable membrane microdomains, with sialylated mucins included in lipid-raft domains (Lantos et al., 2016). It is possible that gp82 and gp90 are differentially partitioned in membrane microdomains of CL and G strains, and the preferential partitioning into rafts could influence their release.

Based on the observation that treatment of G strain MTs with phosphoinositide-specific PLC inhibitor resulted in decreased shedding, of gp82 and gp90, the involvement of PLC is inferred. GPI-solubilizing PLC, detected in G strain, was mostly in a soluble form in MTs and membrane-associated in TCT (de Almeida and Heise, 1993). Soluble PLC would contribute to gp82 and gp90 release by G strain. On the other hand, one possibility for the lack of shedding of these glycoproteins by CL strain is that PLC is mostly membrane associated and segregated from gp82 and gp90 molecules, thus making it unable to exert its enzyme activity. Treatment of CL and G strain MTs with PLC inhibitor was previously found to decrease host cell invasion, possibly because the intracellular Ca^2+^ mobilization required for the internalization process was blocked (Maeda et al., 2012).

Taken together, the present study has contributed to further understand the process of shedding of GPI-anchored surface molecules by *T. cruzi* and reinforced the role played by gp82 and gp90 in regulating MT invasion of host cells.

## Data Availability Statement

The raw data supporting the conclusions of this article will be made available by the authors, without undue reservation.

## Author Contributions

LL, TO, and NY designed the experiments. LL, TO, and JR performed the experiments. NY wrote the manuscript. All authors contributed to the article and approved the submitted version.

## Funding

This work was supported by São Paulo Research Foundation (FAPESP) Grant 2016/15000-4 and Conselho Nacional de Desenvolvimento Científico e Tecnológico (CNPq) Grant 303825/2015-4 and in part by the Coordenação de Aperfeiçoamento de Pessoal de Nível Superior—Brazil (CAPES)—Finance Code 001.

## Conflict of Interest

The authors declare that the research was conducted in the absence of any commercial or financial relationships that could be construed as a potential conflict of interest.

## Publisher’s Note

All claims expressed in this article are solely those of the authors and do not necessarily represent those of their affiliated organizations, or those of the publisher, the editors and the reviewers. Any product that may be evaluated in this article, or claim that may be made by its manufacturer, is not guaranteed or endorsed by the publisher.

## Supplementary Material

Supplementary Figure 1 Higher susceptibility to MT invasion of host cells of larger size. HeLa cells were incubated for 30 min with CL strain MT and processed for immunofluorescence and confocal microscopy visualization of lysosomes (green), nucleus (blue), and gp82 (red). Scale bar = 10 µm. Note the internalized CL strain MT with lysosome membrane marker (white arrow).

Supplementary Figure 2 Protein analysis of CM from CL and G strain MT. Parasites were incubated for 30 min in medium containing 1% FBS. After centrifugation and filtration, the supernatant was analyzed using High Sensitivity Protein 250 kit and the Agilent 2100 Bioanalyzer system.

Supplementary Figure 3 Difference in size between CL and G strain MT. **(A)** Purified MTs were processed for analysis by scanning electron microscopy. Scale-bar = 10 μm. **(B)** The size of nine MTs shown in Figure 4A was evaluated by calculating the area of each parasite, using ImageJ v. 1.53f51, and arbitrary unities were attributed to these areas. The difference between CL and G strain MT was significant (**P* < 0.05).

Supplementary Figure 4 Gp82 and gp90 levels in CM from G strain MT incubated in absence or in the presence of HeLa cells. **(A)** Parasites were placed onto plates, either uncoated or with adherent HeLa cells. After 30 min incubation, the samples were centrifuged, filtered and the supernatant was analyzed by western blotting. **(B)** HeLa cells were incubated for 1 h with CL strain MT in absence or in the presence of CM generated in **(A)**, and the number of internalized parasites was counted. Values are the means ± SD of three independent assays performed in duplicate. The decrease of invasion by CMs was significant (**P* < 0.01).

## References

[B1] Andrews N. W. Hong K. S. Robbins E. S. Nussenzweig V. (1987). Stage-Specific Surface Antigens Expressed During the Morphogenesis of Vertebrate Forms of. Trypanosoma Cruzi Exp. Parasitol. 64, 474–484. doi: 10.1016/0014-4894(87)90062-2 3315736

[B2] Bayer-SantosE.Aguilar-BonavidesC.RodriguesS. P.CorderoE. M.MarquesA. F.Varela-RamirezA.. (2013). Proteomic Analysis of Trypanosoma Cruzi Secretome: Characterization of Two Populations of Extracellular Vesicles and Soluble Proteins. J. Proteome Res. 12 (2), 883–897. doi: 10.1021/pr300947g 23214914

[B3] Borges F. T. Reis L. A. Schor N. (2013). Extracellular Vesicles: Structure, Function, and Potential Clinical Uses in Renal Diseases. Braz. J. Med. Biol. Res. 46, 824–830. doi: 10.1590/1414-431X20132964 24141609 PMC3854311

[B4] Clemente T. M. Cortez C. Novaes A. D. S. Yoshida N. (2016). Surface Molecules Released by Trypanosoma Cruzi Metacyclic Forms Downregulate Host Cell Invasion. PloS Negl. Trop. Dis. 10 (8), e0004883. doi: 10.1371/journal.pntd.0004883 27483135 PMC4970754

[B5] Cortez C. Real F. Yoshida N. (2016). Lysosome Biogenesis/Scattering Increases Host Cell Susceptibility to Invasion by Trypanosoma Cruzi Metacyclic Forms and Resistance to Tissue Culture Trypomastigotes. Cell. Microbiol. 18, 748–760. doi: 10.1111/cmi.12548 26572924 PMC5064668

[B6] Cronemberger-Andrade A. Xander P. Soares R. P. Pessoa N. L. Campos M. A. Ellis C. C.. (2020). *Trypanosoma Cruzi*-Infected Human Macrophages Shed Proinflammatory Extracellular Vesicles That Enhance Host-Cell Invasion via Toll-Like Receptor 2. Front. Cell. Infect. Microbiol. 10. doi: 10.3389/fcimb.2020.00099 PMC7098991 32266161

[B7] de Almeida M. L. Heise N. (1993). Proteins Anchored via Glycosylphosphatidylinositol and Solubilizing Phospholipases in Trypanosoma Cruzi. Biol. Res. 26, 285–312. 7670541

[B8] Fernandes M. C. Cortez M. Flannery A. R. Tam C. Mortara R. A. Andrews N. W. (2011). Trypanosoma Cruzi Subverts the Sphingomyelinase-Mediated Plasma Membrane Repair Pathway for Cell Invasion. J. Exp. Med. 208, 909–921. doi: 10.1084/jem.20102518 21536739 PMC3092353

[B9] Fernandes M. C. Cortez M. Geraldo Yoneyama K. A. Straus A. H. Yoshida N. Mortara R. A. (2007). Novel Strategy in *Trypanosoma Cruzi* Cell Invasion: Implication of Cholesterol and Host Cell Microdomains. Int. J. Parasitol. 37, 1431–1441. doi: 10.1016/j.ijpara.2007.04.025 17582418

[B10] Garcia-Silva M. R. Cura Das Neves R. F. Cabrera-Cabrera F. Sanguinetti J. Medeiros L. C. Robello C.. (2014). Extracellular Vesicles Shed by Trypanosoma Cruzi are Linked to Small RNA Pathways, Life Cycle Regulation, and Susceptibility to Infection of Mammalian Cells. Parasitol. Res. 113, 285–304. doi: 10.1007/s00436-013-3655-1 24241124

[B11] Gonçalves M. F. Umezawa E. S. Katzin A. M. de Souza W. Alves M. J. Zingales B.. (1991). *Trypanosoma Cruzi*: Shedding of Surface Antigens as Membrane Vesicles. Exp. Parasitol. 72, 43–53. doi: 10.1016/0014-4894(91)90119-h 1993464

[B12] Lantos A. B. Carlevaro G. Araoz B. Ruiz Diaz P. Camara M. de los M.. (2016). Sialic Acid Glycobiology Unveils Trypanosoma Cruzi Trypomastigote Membrane Physiology. PloS Pathog. 12 (4), e1005559. doi: 10.1371/journal.ppat.1005559 27058585 PMC4825991

[B13] Maeda F. Y. Cortez C. Alves R. M. Yoshida N. (2012). Mammalian Cell Invasion by Closely Related Trypanosoma Species T. Dionisii and *T. Cruzi* . Acta Tropica 121, 141–147. doi: 10.1016/j.actatropica.2011.10.017 22079376

[B14] Málaga S. Yoshida N. (2001). Targeted Reduction in Expression of Trypanosoma Cruzi Surface Glycoprotein Gp90 Increases Parasite Infectivity. Infect. Immun. 69, 353–359. doi: 10.1128/IAI.69.1.353-359.2001 11119524 PMC97890

[B15] Martins R. M. Alves R. M. Macedo S. Yoshida N. (2011). Starvation and Rapamycin Differentially Regulate Host Cell Lysosome Exocytosis and Invasion by Trypanosoma Cruzi Metacyclic Forms. Cell. Microbiol. 13 (7), 943–954. doi: 10.1111/j.1462-5822.2011.01590.x 21501360

[B16] Mathivanan S. Ji H. Simpson R. J. (2010). Exosomes: Extracellular Organelles Important in Intercellular Communication. J. Proteomics 73, 1907–1920. doi: 10.1016/j.jprot.2010.06.006 20601276

[B17] Nogueira P. M. Ribeiro K. Silveira A. C. O. Campos J. H. Martins-Filho O. A. Bela S. R.. (2015). Vesicles From Different Trypanosoma Cruzi Strains Trigger Differential Innate and Chronic Immune Responses. J. Extracell Vesicles 4:28734. doi: 10.3402/jev.v4.28734 26613751 PMC4662668

[B18] Onofre T. S. Rodrigues J. P. F. Shio M. T. Macedo S. Juliano M. A. Yoshida N. (2021). Interaction of *Trypanosoma Cruzi* Gp82 With Host Cell LAMP2 Induces Protein Kinase C Activation and Promotes Invasion. Front. Cell. Infect. Microbiol. 11. doi: 10.3389/fcimb.2021.627888 PMC7996063 33777840

[B19] Paranaiba L. F. Guarneri A. A. Torrecilhas A. C. Melo M. N. Soares R. P. (2019). Extracellular Vesicles Isolated From *Trypanosoma Cruzi* Affect Early Parasite Migration in the Gut of Rhodnius Prolixus But Not in Triatoma Infestans. Mem. Inst. Oswaldo Cruz 114, e190217. doi: 10.1590/0074-02760190217 31851215 PMC6908325

[B20] Ribeiro K. S. Vasconcellos C. I. Soares R. P. Mendes M. T. Ellis C. C. Aguilera-Flores M.. (2018). Proteomic Analysis Reveals Different Composition of Extracellular Vesicles Released by Two Trypanosoma Cruzi Strains Associated With Their Distinct Interaction With Host Cells. J. Extracell Vesicles 7 (1), 1463779. doi: 10.1080/20013078.2018.1463779 29696081 PMC5912195

[B21] Roberts C. W. McLeod R. Rice D. W. Ginger M. Chance M. L. Goad L. J. (2003). Fatty Acid and Sterol Metabolism: Potential Antimicrobial Targets in Apicomplexan and Trypanosomatid Parasitic Protozoa. Mol. Biochem. Parasitol. 126 (2), 129–142 . doi: 10.1016/S0166-6851(02)00280-3 12615312

[B22] Rodrigues J. P. F. Souza Onofre T. Barbosa B. C. Ferreira É.R. Bonfim-Melo A. Yoshida N. (2019). Host Cell Protein LAMP-2 is the Receptor for Trypanosoma Cruzi Surface Molecule Gp82 That Mediates Invasion. Cell. Microbiol. 21 (5), e13003. doi: 10.1111/cmi.13003 30609224 PMC6590364

[B23] Rodrigues J. P. F. Takahashi Sant’ana G. H. Juliano M. A. Yoshida N. (2017). Inhibition of Host Cell Lysosome Spreading by Trypanosoma Cruzi Metacyclic Stage-Specific Surface Molecule Gp90 Downregulates Parasite Invasion. Infect. Immun. 85 (9), e00302–17. doi: 10.1128/IAI.00302-17 28607099 PMC5563561

[B24] Souto-Padrón T. de Souza W. (1983). Freeze-Fracture Localization of Filipin-Cholesterol Complexes in the Plasma Membrane of Trypanosoma Cruzi. J. Parasitol. 69, 129–137. doi: 10.2307/3281287 6402578

[B25] Tardieux I. Webster P. Ravesloot J. Boron W. Lunn J. A. Heuser J. E.. (1992). Lysosome Recruitment and Fusion are Early Events Required for Trypanosome Invasion of Mammalian Cells. Cell 71, 1117–1130. doi: 10.1016/s0092-8674(05)80061-3 1473148

[B26] Teixeira M. M. Yoshida N. (1986). Stage-Specific Surface Antigens of Metacyclic Trypomastigotes of Trypanosoma Cruzi Identified by Monoclonal Antibodies. Mol. Biochem. Parasitol. 18, 271–282. doi: 10.1016/0166-685(86)90085-x 3515178

[B27] Torrecilhas A. C. Soares R. P. Schenkman S. Fernández-Prada C. Olivier M. (2020). Extracellular Vesicles in Trypanosomatids: Host Cell Communication. Front. Cell. Infect. Microbiol. 10:602502. doi: 10.3389/fcimb.2020.602502 33381465 PMC7767885

[B28] Torrecilhas A. C. Tonelli R. R. Pavanelli W. R. da Silva J. S. Schumacher R. I. de Souza W.. (2009). *Trypanosoma Cruzi*: Parasite Shed Vesicles Increase Heart Parasitism and Generate an Intense Inflammatory Response. Microbes Infect. 11, 29–39. doi: 10.1016/j.micinf.2008.10.003 19028594

[B29] von Tresckow B. Kallen Strandmann E. P. Borchmann P. Lange H. Engert A.. (2004). )Depletion of Cellular Cholesterol and Lipid Rafts Increases Shedding of CD30. J. Immunol. 172, 4324–4331. doi: 10.4049/JIMMUNOL.172.7.4324 15034047

[B30] Wyllie M. P. Ramirez M. I. (2017). Microvesicles Released During the Interaction Between *Trypanosoma Cruzi* TcI and TcII Strains and Host Blood Cells Inhibit Complement System and Increase the Infectivity of Metacyclic Forms of Host Cells in a Strain-Independent Process. Pathog. Dis. 75 (7), ftx077. doi: 10.1093/femspd/ftx077 28859399

[B31] Yáñez-Mó M. Siljander P. R. M. Andreu Z. Zavec A. B. Borràs F. E. Buzas E. I.. (2015). Biological Properties of Extracellular Vesicles and Their Physiological Functions. J. Extracell Vesicles 4, 1–60. doi: 10.3402/jev.v4.27066 PMC4433489 25979354

[B32] Yoshida N. (2006). Molecular Basis of Mammalian Cell Invasion by Trypanosoma Cruzi. An Acad. Bras. Cienc. 78, 87–111. doi: 10.1590/S0001-37652006000100010 16532210

[B33] Zaborowski M. P. Balaj L. Breakefield X. O. Lai C. P. (2015). Extracellular Vesicles: Composition, Biological Relevance, and Methods of Study. Bioscience 65, 783–797. doi: 10.1093/biosci/biv084 26955082 PMC4776721

[B34] Zingales B. Andrade S. G. Briones M. R. S. Campbell D. A. Chiari E. Fernandes O.. (2009). A New Consensus for Trypanosoma Cruzi Intraspecific Nomenclature: Second Revision Meeting Recommends TcI to TcVI. Mem. Inst. Oswaldo Cruz 104, 1051–1054. doi: 10.1590/50074-0276009000700021 20027478

