# Supplementary figures and images for: Shedding of Trypanosoma cruzi Surface Molecules That Regulate Host Cell Invasion Involves Phospholipase C and Increases Upon Sterol Depletion
Source: Front Cell Infect Microbiol. 2021 Oct 19;11:769722. doi: 10.3389/fcimb.2021.769722 (PMC8560688; doi:10.3389/fcimb.2021.769722)

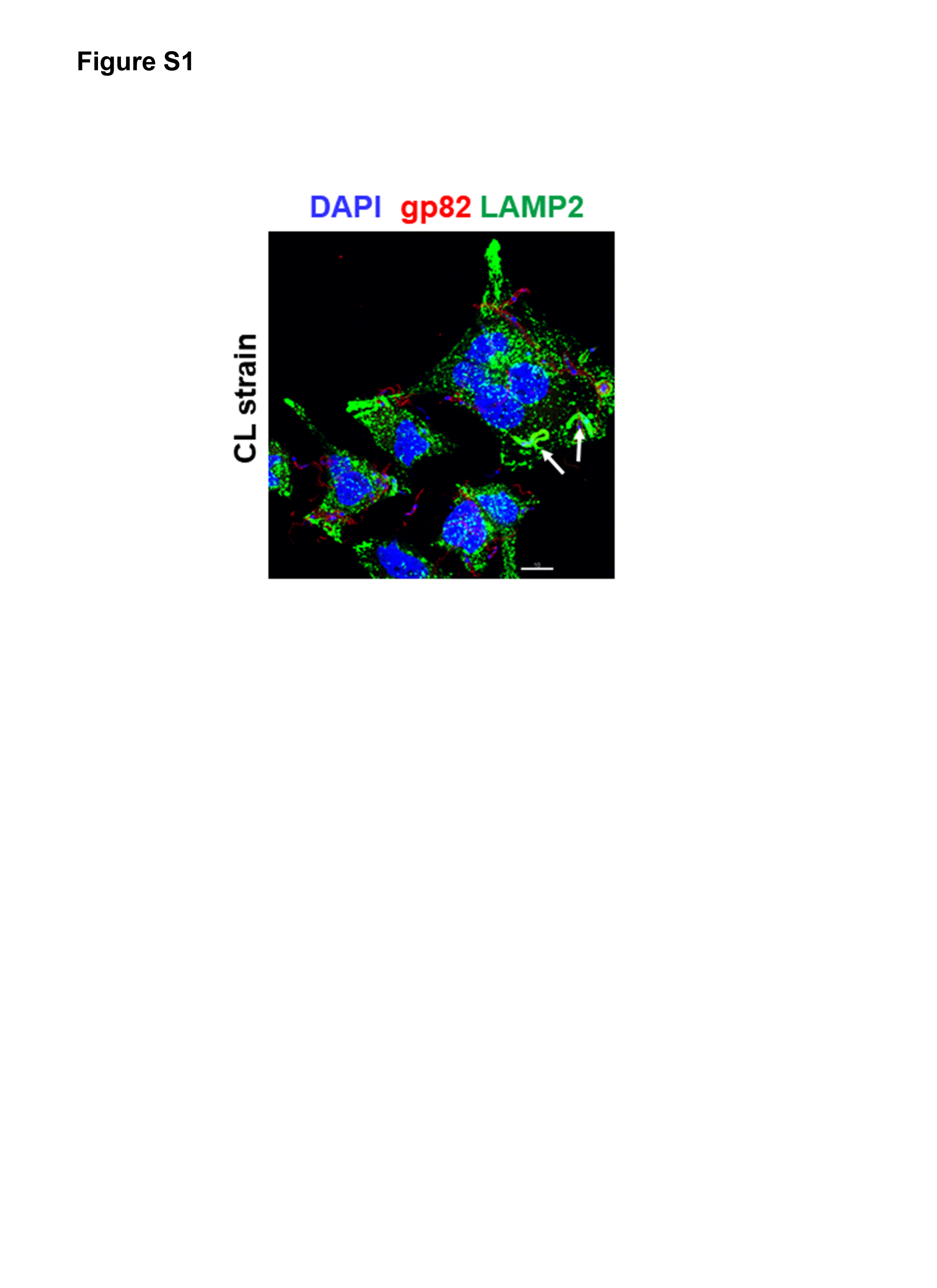

Supplement: Supplementary Figure 1 — Higher susceptibility to MT invasion of host cells of larger size. HeLa cells were incubated for 30 min with CL strain MT and processed for immunofluorescence and confocal microscopy visualization of lysosomes (green), nucleus (blue), and gp82 (red). Scale bar = 10 µm. Note the internalized CL strain MT with lysosome membrane marker (white arrow). [file Image_1.tif]

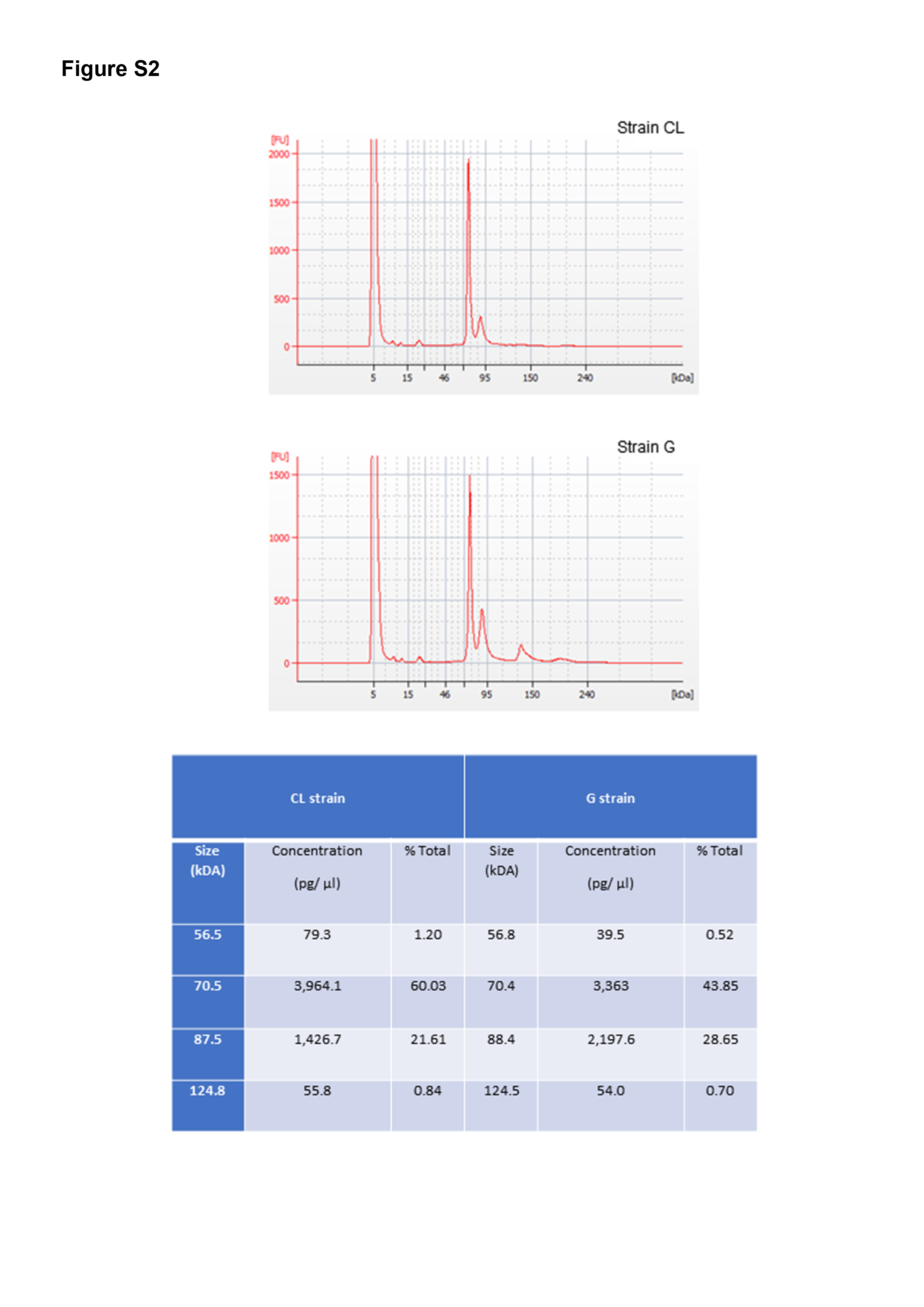

Supplement: Supplementary Figure 2 — Protein analysis of CM from CL and G strain MT. Parasites were incubated for 30 min in medium containing 1% FBS. After centrifugation and filtration, the supernatant was analyzed using High Sensitivity Protein 250 kit and the Agilent 2100 Bioanalyzer system. [file Image_2.tif]

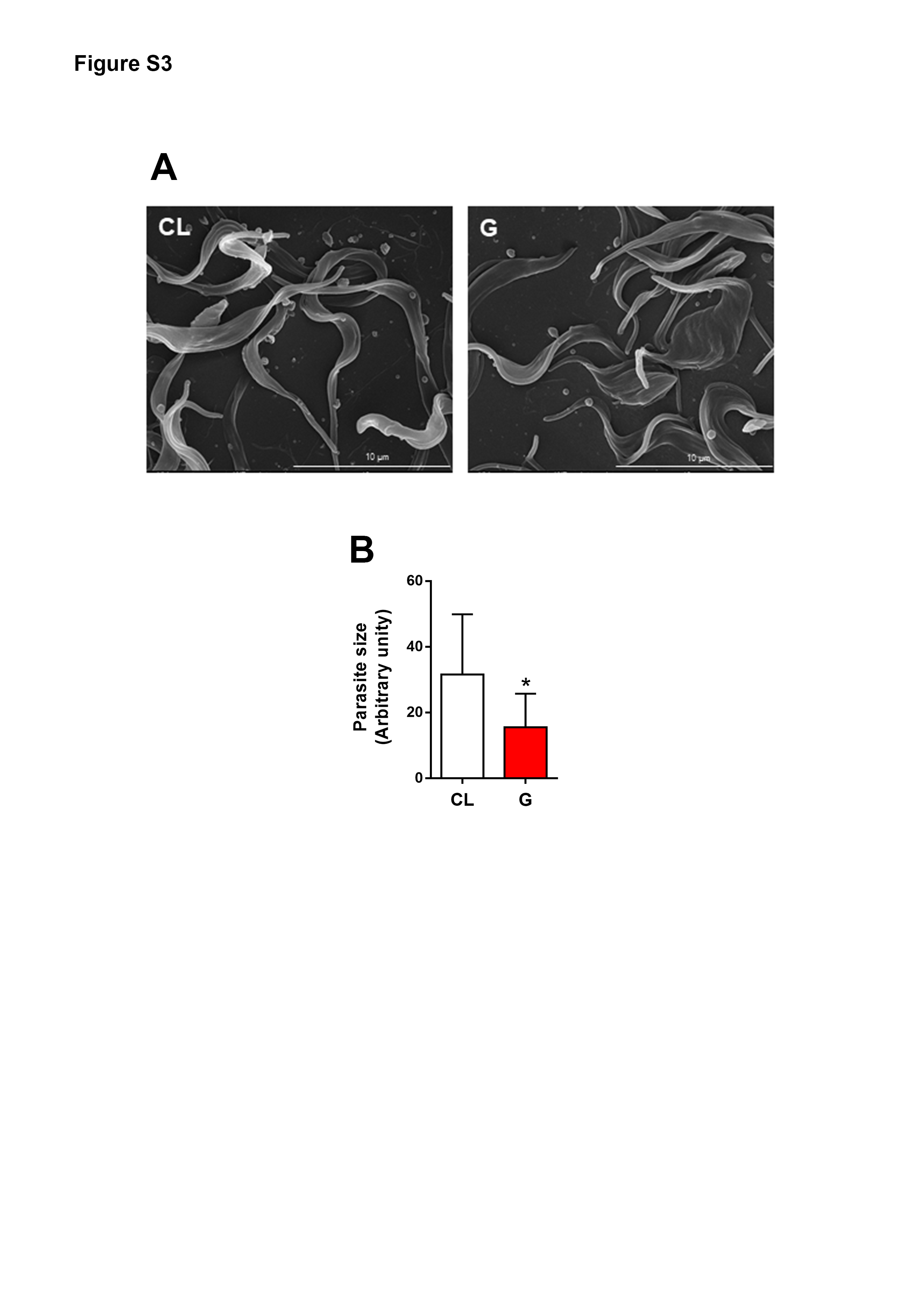

Supplement: Supplementary Figure 3 — Difference in size between CL and G strain MT. (A) Purified MTs were processed for analysis by scanning electron microscopy. Scale-bar = 10 μm. (B) The size of nine MTs shown in Figure 4A was evaluated by calculating the area of each parasite, using ImageJ v. 1.53f51, and arbitrary unities were attributed to these areas. The difference between CL and G strain MT was significant (*P < 0.05). [file Image_3.tif]

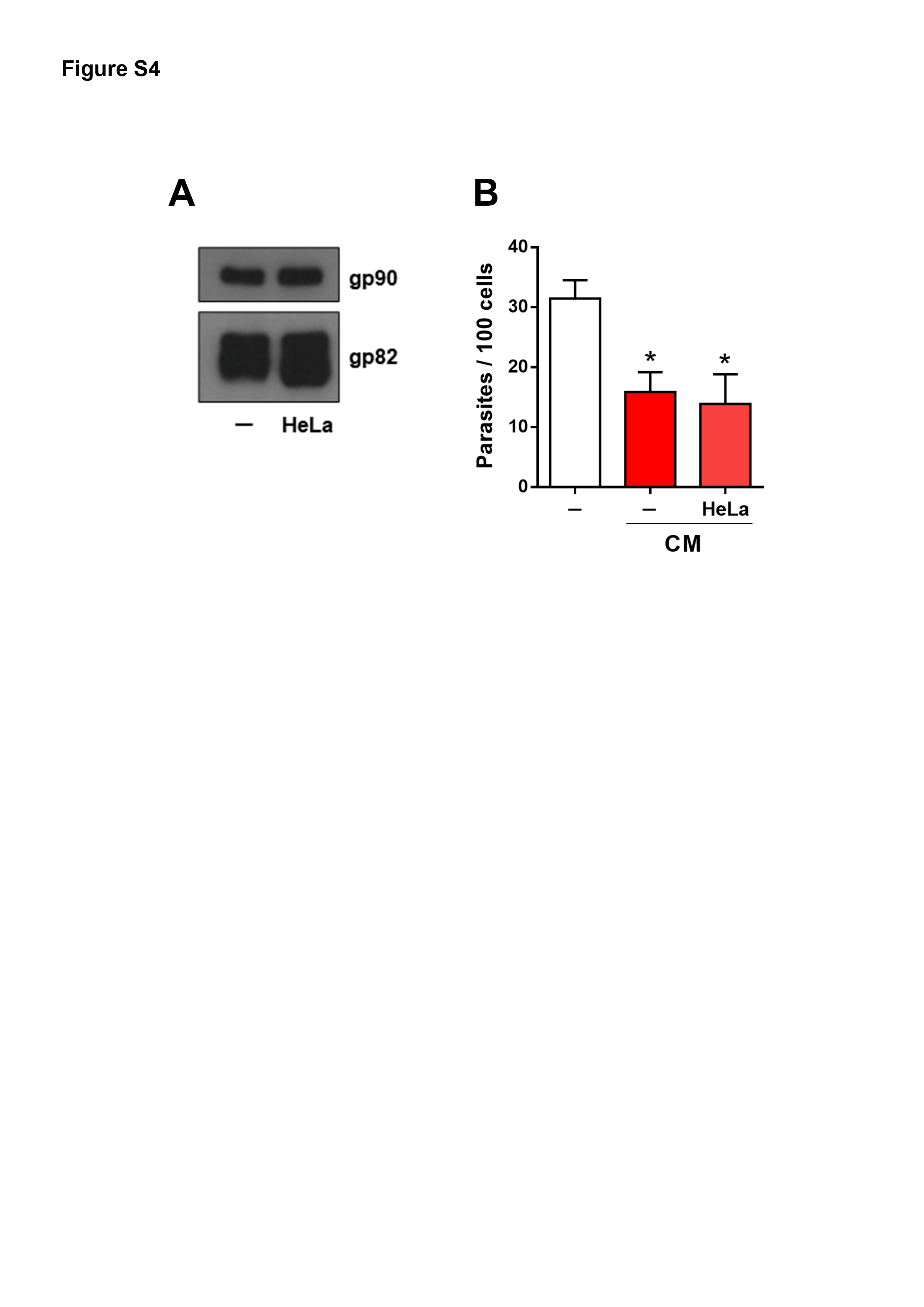

Supplement: Supplementary Figure 4 — Gp82 and gp90 levels in CM from G strain MT incubated in absence or in the presence of HeLa cells. (A) Parasites were placed onto plates, either uncoated or with adherent HeLa cells. After 30 min incubation, the samples were centrifuged, filtered and the supernatant was analyzed by western blotting. (B) HeLa cells were incubated for 1 h with CL strain MT in absence or in the presence of CM generated in (A), and the number of internalized parasites was counted. Values are the means ± SD of three independent assays performed in duplicate. The decrease of invasion by CMs was significant (*P < 0.01). [file Image_4.tif]
